# Differences in Prevalence and Severity of Liver Disease Between Lateral Tunnel and Extracardiac Conduit Fontan

**DOI:** 10.1016/j.jacadv.2026.102680

**Published:** 2026-03-17

**Authors:** Daniella Haas, Mohamed Ellabbad, William R. Miranda, Moira B. Hilscher, Heidi M. Connolly, Alexander C. Egbe

**Affiliations:** a Mayo Medical School, Minnesota, USA; b Department of Cardiovascular Medicine, Minnesota, USA; c Division of Gastroenterology and Hepatology, Mayo Clinic Rochester, Minnesota, USA

**Keywords:** biomarkers, Fontan physiology, liver disease, risk stratification

## Abstract

**Background:**

Recent studies suggest differences in liver disease severity based on the type of total cavopulmonary connection (TCPC).

**Objectives:**

The purpose of this study was to compare the severity and progression of liver disease between patients with extracardiac conduit (ECC) vs lateral tunnel (LT) using 2 separate cohorts: i) TCPC at the initial Fontan operation (primary-TCPC cohort) and ii) Fontan conversion (FC) to TCPC (FC-TCPC cohort).

**Method:**

This is a retrospective study of adults with TCPC (ECC [N = 62, 46%]; LT [N = 73,54%). Liver disease severity was assessed at baseline using biomarkers (model for end-stage liver disease excluding international normalized ratio, Fibrosis-4 [FIB-4], aspartate aminotransferase to platelet ratio index [APRI]), and progression of liver disease was assessed as temporal change in biomarkers at 3, 5, and 7 years.

**Results:**

In the primary-TCPC cohort (ECC [N = 62,46%]; LT [N = 73,54%), patients with ECC had a higher prevalence of cirrhosis (27% vs 12%; *P* = 0.03) and worse disease severity at baseline (APRI 0.46 [0.34;0.61] vs 0.39 [0.29;0.52], *P* < 0.001; FIB-4 (0.89 [0.59;1.12] vs 0.76 [0.31;0.99], *P* = 0.009) compared to LT. Both groups had progression of liver disease (temporal increase in biomarker levels), but the ECC group had a greater temporal increase in biomarker levels resulting in a higher relative increase in model for end-stage liver disease excluding international normalized ratio, FIB-4, and APRI. There were no significant differences in prevalence and severity of liver disease at baseline, or liver disease progression during follow-up between patients with ECC vs LT in the FC-TCPC group.

**Conclusions:**

ECC was associated with a higher prevalence and worse severity of liver disease at baseline, and greater disease progression during follow-up compared to those with LT. Further studies are required to determine the optimal strategies for the management of liver disease in patients with ECC.

Fontan-associated liver disease (FALD) is common in adults with Fontan palliation, and it is associated with increased morbidity and mortality.^1^, ^2^, ^3^, ^4^ The pathogenesis of FALD is complex, but it is primarily driven by hemodynamic factors since high central venous pressure is inherent in Fontan physiology.^5^ Chronic exposure to high central venous pressure results in structural and functional remodeling of the liver parenchyma leading to hepatocyte necrosis and fibrosis.^3^^,^^6^^,^^7^ FALD is typically subclinical for several decades despite extensive structural changes in the liver architecture.^1^^,^^3^ However, some patients will eventually present with features of portal hypertension (esophageal varices, ascites, and splenomegaly), liver cirrhosis, or hepatocellular carcinoma.^8^, ^9^, ^10^, ^11^ The conventional paradigm for the pathogenesis of FALD has centered on Fontan hemodynamics (high central venous pressure) rather than the type of Fontan connection since abnormal Fontan hemodynamics rather than the type of Fontan connection is postulated to be the primary determinant of FALD.^3^

The total cavopulmonary connection (TCPC) has been the standard of care for the Fontan operation for more than 3 decades, and the specific type of TCPC (lateral tunnel [LT] vs extracardiac conduit [ECC]) offered to a patient is typically determined by institutional preference and expertise as the long-term outcomes of both techniques are largely comparable.^12^, ^13^, ^14^ However, recent studies suggest that patients with ECC, especially those with smaller Fontan conduit, had a higher prevalence of cirrhosis compared to those with LT.^15^^,^^16^ However, it is unknown whether these differences in structural remodeling of the liver between ECC and LT translate to differences in liver function.^6^ The current study aims to address this knowledge gap. Our study objectives were to: 1) compare the prevalence of cirrhosis and liver biomarkers at baseline encounter between patients with ECC vs LT; and 2) compare temporal change in liver biomarkers between patients with ECC vs LT.

## Methods

### Study population

This is a retrospective cohort study of adults (age ≥18 years) with TCPC who received care at Mayo Clinic between January 1, 2003, and December 31, 2024. The patients were identified through the MACHD (Mayo Adult Congenital Heart Disease) registry, and the Mayo Clinic Institutional Review Board approved this study.^17^ The study inclusion criteria were: 1) ≥2 measurements of comprehensive metabolic panel (CMP) and complete blood count (CBC) in the outpatient clinic with ≥2 years between measurements; 2) liver imaging (abdominal ultrasound or cross-sectional imaging); and 3) evaluation in the hepatology clinic. We divided the study population into 2 cohorts. The first cohort comprised of patients who received TCPC at the time of initial Fontan operation (primary-TCPC cohort), while the second cohort comprised of patients with atriopulmonary connection who underwent Fontan conversion (FC) to TCPC (FC-TCPC cohort). We defined TCPC as LT vs ECC, and the patients who received intra-atrial conduit were classified as ECC.

### Data collection

#### Primary-TCPC cohort

The first outpatient encounter during which CBC and CMP were measured was considered as the baseline encounter. The medical records were reviewed, and clinical data (clinical assessments, imaging, cardiac catheterization, and laboratory data) obtained within 12 months of the baseline encounter were used to define the baseline characteristics of the cohort.

#### FC-TCPC cohort

The first outpatient encounter after FC operation during which CBC and CMP were measured was considered as the baseline encounter, and the medical records obtained within 12 months of the baseline encounter were used to define the baseline characteristics of the cohort.

### Study outcomes

#### Cirrhosis

For the purpose of this study, we defined cirrhosis as the presence of imaging or histologic evidence of cirrhosis plus clinical features identified by a hepatologist with experience in FALD.^4^^,^^15^ We defined imaging evidence of cirrhosis as the presence of coarse echotexture and nodular contour based on liver ultrasound or cross-sectional imaging, and histologic evidence of cirrhosis as stage 3 or 4 portal or sinusoidal fibrosis on biopsy.^1^^,^^18^

#### Liver biomarkers

We assessed liver disease severity using 3 liver biomarker scores calculated using the components of CMP and CBC measured at each clinic visit. 1) Model for end-stage liver disease excluding international normalized ratio (MELD-XI) score was calculated as: 5.11× ln (serum total bilirubin in mg/dL) + 11.76 × ln (serum creatinine in mg/dL) + 9.44.^19^ In order to avoid negative scores, a lower limit of total bilirubin and creatinine was set at 1.0 mg/dL.^6^^,^^20^ 2) Fibrosis-4 (FIB-4) score was calculated as age [years] x aspartate aminotransferase [U/L]/platelet count [10^9^/L] x √alanine transaminase [U/L]. 3) Aspartate aminotransferase to platelet ratio index (APRI) was calculated as: 100 × (aspartate aminotransferase [U/L]/40 ÷ platelet count [10^9^/L]).^6^^,^^20^

The liver biomarker scores (MELD-XI, FIB-4, and APRI) were calculated at the baseline encounter (time 0), and at 3 (2-4), 5 (4-6), and 7 (6-8) years from the baseline encounter. The temporal change in liver biomarker scores at each encounter was calculated as relative change from baseline. For instance, temporal change in MELD-XI 3 years was calculated as follows: relative Δ_MELD-XI = ([MELD-XI at 3y MELD-XI at baseline] ÷ MELD-XI at baseline) ×100. Positive values signify temporal increase in liver biomarkers consistent with worsening disease severity, while negative values signify temporal decrease in liver biomarkers consistent with improvement in disease severity.

### Statistical analysis

Data were presented as mean ± SD, median (IQR), and count (%). Between-group comparisons were performed using unpaired *t*-test, Wilcoxon rank sum test, and Fisher exact test, as appropriate. Temporal changes in liver biomarkers were assessed using paired *t*-test. Separate analyses were performed for the primary-TCPC and FC-TCPC cohorts for all study outcomes. Missing data were handled using conditional imputation. All statistical analyses were performed with BlueSky Statistics software (version. 7.10; BlueSky Statistics LLC) and JMP statistical software (version 17.1.0, JMP Statistical Discovery LLC). *P* value < 0.05 was considered to be statistically significant for all analyses.

## Results

### Baseline characteristics

The study comprised 135 patients in the primary-TCPC cohort and 104 patients in the FC-TCPC cohort. Of the 135 patients in the primary-TCPC cohort, 73 (54%) received LT, while 62 (46%) received ECC. The patients with LT were older at the baseline encounter (26 [22;30] vs 20 [18;24] years; *P* = 0.002), had a longer duration of Fontan physiology (23 [19;26] vs 17 [15;20] years; *P* < 0.001), were more likely to have systemic morphologic left ventricle (60% vs 47%; *P* = 0.04), and had a higher prevalence of atrial arrhythmias (34% vs 16%; *P* = 0.02), compared to those with ECC. There were no other significant between-group differences in comorbidities and hemodynamic indices for patients with LT vs ECC (Table 1).

**Table 1:** Baseline Characteristics

	Primary TCPC (N = 135)		FC-TCPC (N = 104)	
	LT (n = 73, 54%)	ECC (n = 62, 46%)	LT (n = 19, 18%)	ECC (n = 85, 82%)
Age, y	26 (22;30)^a^	20 (18;24)^a^	26 (20;35)	27 (20;34)
Male	39 (53%)	31 (52%)	11 (58%)	54 (64%)
Body mass index, kg/m^2^	23.4 (20.9;27.3)	22.7 (20.3;26.7)	24.1 (21.0;26.5)	24,3 (21.2;27.5)
Systemic oxygen saturation, %	92 (90;94)	92 (90;94)	92 (90;94)	93 (90;95)
Age at Fontan operation, y	3 (2;5)	3 (3;6)	5 (3;10)	5 (3;8)
Duration of Fontan physiology, y	23 (19;26)	17 (15;20)^a^	21 (17;28)	22 (18;29)
Interval from FC	---	---	3 (1,5)	3 (1, 4)
Anatomy				
Heterotaxy	2 (3%)	3 (5%)	1 (5%)	3 (4%)
Ventricular morphology				
LV	44 (60%)^a^	29 (47%)^a^	17 (90%)	76 (89%)
RV	24 (33%)	32 (52%)	1 (5%)	7 (8%)
Mixed/indeterminate	5 (7%)	1 (2%)	1 (5%)	2 (2%)
Comorbidities				
Atrial arrhythmias	25 (34%)^a^	10 (16%)^a^	13 (68%)	48 (57%)
Atrial flutter/tachycardia	15 (21%)	7 (11%)	9 (47%)	35 (41%)
Atrial fibrillation	6 (8%)	4 (7%)	6 (32%)	25 (29%)
CKD III-V	4 (6%)	1 (2%)	2 (11%)	7 (8%)
Protein losing enteropathy	4 (6%)	6 (10%)	3 (16%)	6 (7%)
Medications				
Diuretics	27 (37%)	15 (24%)	10 (53%)	50 (59%)
Beta-blockers	37 (51%)^a^	20 (32%)^a^	7 (37%)	35 (41%)
ACEI/ARB	50 (69%)	43 (70%)	10 (53%)	55 (65%)
MRA	16 (22%)	16 (26%)	5 (26%)	23 (27%)
Echocardiographic indices				
Ventricular EDV index, mL/m^2^	95 (65;112)	92 (69;117)	87 (69;109)	83 (62;107)
Ventricular ESV index, mL/m^2^	40 (31;59)	43 (35;52)	37 (32;52)	34 (27;50)
Ventricular EF, %	50 ± 12	50 ± 9	53 ± 9	55 ± 8
Ventricular SV index, mL/m^2^	44 ± 12	57 ± 13	46 ± 14	46 ± 16
≥Mod systemic AVV regurgitation	1 (2%)	5 (8%)	1 (5%)	2 (2%)
Cardiac MRI	N = 41	N = 45	N = 12	N = 58
Ventricular EDV index, mL/m^2^	96 (64;117)	87 (57;94)	85 (39;105)	82 (53;96)
Ventricular ESV index, mL/m^2^	49 (33;72)	39 (30;51)	43 (26;50)	41 (20;42)
Ventricular SV index, mL/m^2^	42 ± 17	39 ± 16	34 ± 21	41 ± 17
Ventricular EF, %	49 ± 10	48 ± 10	47 ± 11	56 ± 11
Cardiac catheterization data	N = 49	N = 44	N = 15	N = 71
Fontan pressure, mm Hg	13.2 ± 3.1	12.7 ± 3.0	14.7 ± 3.6	15.2 ± 4.3
PAWP, mm Hg	9.2 ± 2.6	8.9 ± 2.8	9.9 ± 3.9	10.9 ± 4.0
PVR index, WU∗m^2^	1.87 ± 1.01	1.91 ± 1.66	2.42 ± 0.71	2.39 ± 0.82
Cardiac index, l/min/m^2^	2.34 ± 1.01	2.41 ± 1.35	2.26 ± 1.18	2.33 ± 1.04

ACEI/ARB = angiotensin-converting enzyme inhibitor/angiotensin-II receptor blocker; AVV = atrioventricular valve; CKD = chronic kidney disease; ECC = extracardiac conduit; EF = ejection fraction; EDV = end-diastolic volume; ESV = end-systolic volume; FC = Fontan conversion; LV = left ventricle; LT = lateral tunnel; MRA = mineralocorticoid receptor antagonist; MRI = magnetic resonance imaging; RV = right ventricle; PAWP = pulmonary artery wedge pressure; PVR = pulmonary vascular resistance; SV = stroke volume; TCPC = total cavopulmonary connection; WU = Woods Unit.

Values are mean ± SD, median (Q1;Q3), or n (%). Separate between-group comparisons were performed for LT vs ECC for primary TCPC cohort and the FC-TCPC cohort.

a Signify statistically significant differences (LT vs ECC) within each cohort.

Of 104 patients in the FC-TCPC group, 85 (82%) received ECC, while 19 (18%) received LT. Both groups had similar age at the initial Fontan operation (5 [3-8] years for ECC vs 5 [3;10] years for LT; *P* = 0.79), age at the time of FC (25 [19;32] years for ECC vs 24 [19;31] years for LT; *P* = 0.66), and age at the baseline encounter (27 [20;34] years for ECC vs 26 [20;35] years for LT; *P* = 0.46) (Table 1).

### Prevalence of cirrhosis and liver biomarkers at baseline

Among the 135 patients in the primary-TCPC cohort, patients with ECC had a higher prevalence of liver cirrhosis at the baseline encounter compared to those with LT (27% vs 12%; *P* = 0.03, respectively). The patients with ECC also had worse liver biomarker scores at baseline encounter compared to those with LT, as evidenced by higher APRI (0.46 [0.34;0.61] vs 0.39 [0.29;0.52], *P* < 0.001, respectively) and FIB-4 (0.89 [0.59;1.12] vs 0.76 [0.31;0.99], *P* = 0.009, respectively) scores (Table 2). There was no significant between-group difference in MELD-XI for ECC vs LT subgroups (Table 2). Among the ECC group with available data for Fontan conduit size (N = 43), the mean conduit size was 18 ± 2 mm. There was correlation between conduit size and APRI (r = -0.32; *P* = 0.006), FIB-4 (r = 0.29; *P* = 0.01), but nor MELD-XI score (r = 0.27; *P* = 0.09).

**Table 2:** Liver Data

	Primary TCPC (N = 135)		FC-TCPC (N = 104)	
	LT (n = 73, 54%)	ECC (n = 62, 46%)	LT (n = 19, 18%)	ECC (n = 85, 82%)
Baseline liver biomarker scores				
MELD-XI	10.2 (9.4;12.5)	10.6 (9.4;12.3)	11.6 (9.8;13.1)	11.9 (9.6; 13.7)
APRI	0.39 (0.29;0.52)^a^	0.46 (0.34;0.61)^a^	0.48 (0.29;0.63)	0.45 (0.32;0.65)
FIB-4	0.76 (0.31;0.99)^a^	0.89 (0.59;1.12)^a^	0.97 (0.66;1.43)	0.95 (0.61; 1.36)
Cirrhosis diagnosis				
Based on hepatologist evaluation	9 (12%)^a^	17 (27%)^a^	6 (32%)	26 (31%)
Imaging diagnosis	11 (16%)^a^	19 (31%)^a^	6 (32%)	28 (33%)
Histologic diagnosis	2 (8%) [n = 26]	4 (17%) [n = 23]	2 (29%) [n = 7]	7 (26%) [n = 26]
VAST score ≥2	6 (12%) [n = 61]	11 (20%) [n = 56]	6 (25%) [n = 17]	25 (33%) [n = 76]

APRI = aspartate aminotransferase to platelet ratio index; FIB-4 = Fibrosis-4; MELD-XI = model of end-stage liver disease excluding international normalized ratio; VAST = varices, ascites, splenomegaly, thrombocytopenia; and other abbreviations as in Table 1.

Values are median (Q1;Q3) or n (%). Separate between-group comparisons were performed for LT vs ECC for primary TCPC cohort and the FC-TCPC cohort.

a Signify statistically significant differences (LT vs ECC) within each cohort. [N] Signify number of patients with available data.

### Temporal change in liver biomarkers

Among patients in the primary-TCPC cohort, we observed a temporal increase in all liver biomarkers from baseline through Y7 in the ECC group (MELD-XI [*P* trend < 0.001], APRI [*P* trend = 0.007], and FIB-4 [*P* trend < 0.001]) as well as for FIB-4 (*P* trend = 0.002) in the LT group. Central Illustration shows the relative increase in liver biomarkers from baseline measurement at different time points in primary-TCPC cohort. Compared to the LT group, the ECC group had greater temporal increase in MELD-XI at Y7 (relative_Δ 10.6% [95% CI: 7.9-13.7] vs 6.8% [95% CI: 2.9-10.1], *P* = 0.02), FIB-4 at Y7 (relative_Δ 9.9% [95% CI: 6.8-12.2] vs 6.7% [95% CI: 4.7;8.5], *P* = 0.007), and APRI at Y5 (relative_Δ 9.8% [95% CI: 6.7-12.6] vs 4.9% [95% CI: 2.9-7.8], *P* < 0.001) and at Y7 (relative_Δ 12.2% [95% CI: 9.6-14.8] vs 7.1% [95% CI: 5.0-9.4], *P* = 0.01) (Central Illustration).

**Central Illustration:**
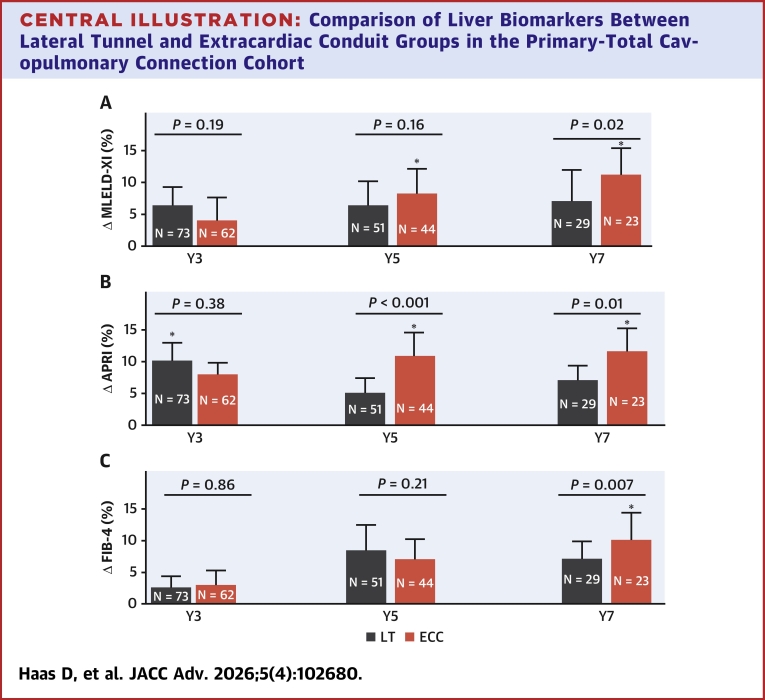
**Comparison of Liver Biomarkers Between Lateral Tunnel and Extracardiac Conduit Groups in the Primary-Total Cavopulmonary Connection Cohort** Bar charts plot comparing temporal change in biomarkers (expressed as relative_Δ from baseline [95% CI]) at different time points between patients with lateral tunnel (black) vs extracardiac conduit (red) in the primary-total cavopulmonary connection cohort. Liver biomarker assessment was based on the model for end-stage liver disease (MELD-XI) (A), aspartate aminotransferase to platelet ratio index (APRI) (B), and Fibrosis-4 (FIB-4) (C). ∗Statistically significant increase from baseline. *P* values were derived from comparisons of temporal change in biomarkers between lateral tunnel vs extracardiac conduit. Primary-total cavopulmonary connection cohort comprise of patients who underwent total cavopulmonary connection at the time of initial Fontan operation. Abbreviations as in Figure 1.

**Figure 1:**
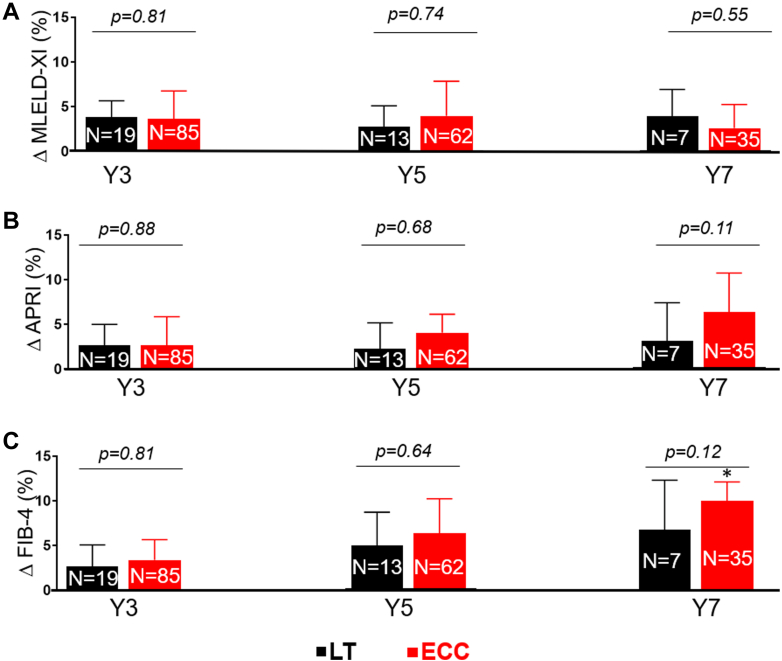
**Comparison of Liver Biomarkers Between Lateral Tunnel and Extracardiac Conduit Groups in the Fontan Conversion-Total Cavopulmonary Connection Cohort** Bar charts plot comparing temporal change in biomarkers (expressed as relative_Δ from baseline [95% CI]) at different time points between patients with lateral tunnel (black) vs extracardiac conduit (red) in the Fontan conversion-total cavopulmonary connection cohort. Liver biomarker assessment was based on the model for end-stage liver disease (MELD-XI) (A), aspartate aminotransferase to platelet ratio index (APRI) (B), and Fibrosis-4 (FIB-4) (C). ∗Statistically significant increase from baseline. *P* values were derived from comparisons of temporal change in biomarkers between lateral tunnel vs extracardiac conduit. Primary-total cavopulmonary connection cohort comprise of patients who underwent total cavopulmonary connection at the time of initial Fontan operation. APRI = aspartate aminotransferase to platelet ratio index; ECC = extracardiac conduit; FIB-4 = fibrosis-4; LT = lateral tunnel; MELD-XI = model for end-stage liver disease excluding international normalized ratio.

Among the patients in the FC-TCPC cohort, we observed a temporal increase from baseline through Y7 in APRI (*P* trend = 0.002) and FIB-4 (*P* trend < 0.001) but not MELD-XI (*P* trend = 0.44) in the ECC group. We also observed a temporal increase from baseline through Y7 in FIB-4 (*P* trend < 0.001) but not APRI (*P* trend = 0.31) and MELD-XI (p trend 0.42) in the LT group. Figure 1 shows the relative increase in liver biomarkers from baseline measurement at different time points in the FC-TCPC cohort. There was no significant between-group difference in temporal change in liver biomarkers (Figure 1).

## Discussion

In this study, we compared structural and functional remodeling of the liver between patients with ECC vs LT using 2 separate cohorts with different characteristics. The main findings were as follows. 1) In the primary-TCPC cohort, patients with ECC had a higher prevalence of liver cirrhosis and worse liver biomarkers at baseline, as well as a greater deterioration in liver function as evidenced by a greater temporal increase in liver biomarkers during follow-up compared to patients with LT. Additionally, we observed that smaller conduit size correlated with worse liver function (higher liver biomarker scores) within the ECC group. 2) In contrast, we did not observe any significant differences in the prevalence and severity of liver disease at baseline, or differences in liver disease progression during follow-up between patients with ECC vs LT in the FC-TCPC group.

Elevated central venous pressure, an inherent hemodynamic limitation of the Fontan physiology, results in chronic changes in the structure and function of the liver leading to FALD.^3^^,^^5^^,^^6^^,^^21^^,^^22^ As a result, the prevalence and severity of FALD correlate with older age and worse Fontan hemodynamics (elevated Fontan pressures).^5^^,^^6^ These factors, in turn, influence management, whereby patients with FALD and elevated Fontan pressures are screened for reversible causes of high Fontan pressures such as Fontan pathway stenosis and pulmonary vascular disease.^23^ In a recent study, Kisamori et al observed a higher prevalence of cirrhosis at 15 years post-Fontan operation in patients with ECC compared with LT (30% vs 4%) based on a large cohort of 328 patients with TCPC suggesting that ECC may be associated with a higher risk of FALD.^15^ These findings are consistent with results of a prior study by Evan et al showing a higher liver fibrosis score in patients with ECC compared to LT.^16^ While Kisamori et al study was based on a large cohort, its limitations include lack of liver imaging data in more than half of the cohort, indices of liver function were not presented, and liver disease progression was not assessed.^15^ The current study builds on this foundation while addressing these limitations. First, all patients included in the current study had multiparametric liver evaluation (imaging, biomarker assay, and clinical assessment by hepatologist), which in turn, minimized selection bias. Second, the availability of serial biomarker assay in the primary-TCPC cohort demonstrated a greater progression of liver disease in patients with ECC compared to LT, despite a younger age in the ECC group, and similar hemodynamic indices between the 2 groups. Furthermore, the LT group had worse liver disease biomarkers compared to the ECC group in the primary-TCPC cohort, despite having a short duration of Fontan physiology. Collectively, this suggests that the greater disease progression (higher temporal increase in liver biomarkers) observed in the ECC group was likely related to inherent characteristics of the ECC rather than differences in patient characteristics and Fontan hemodynamics. Previous studies suggest that the rigid circumferential conduit in ECC results in increased resistance leading to energy loss and worse hemodynamics, in contrast to the more compliant tissue characteristics of LT or atriopulmonary connection.^15^^,^^16^^,^^24^ Third, the absence of a difference in the prevalence, severity, and progression of liver disease between ECC and LT in the FC-TCPC cohort provides additional insight into effect of duration of exposure (ie, duration of ECC) on subsequent liver disease progression. The median interval from FC to baseline liver evaluation was only 2 years, which may not be long enough to detect differences in functional remodeling of the liver between the ECC and LT groups. Consistent with this postulate, the ECC group showed a trend toward a higher temporal increase in APRI and FIB-4 at 7 years although these changes did not achieve statistical significance.

## Clinical implications and future directions

The results of the current study have important clinical implications such as in deciding type of Fontan connection at the time of initial Fontan operation. While the superiority of TCPC over atriopulmonary connection has been empirically validated in multiple studies, the data from studies comparing outcomes of ECC vs LT are somewhat conflicting.^12^, ^13^, ^14^ The benefits of ECC include lower risk of atrial arrhythmias and thromboembolic complications, while the benefits of LT include lower risk of reoperation/reintervention.^12^, ^13^, ^14^ The results of the current study, which are consistent with recent studies,^15^^,^^16^ suggest that lower risk of liver disease may be an additional benefit of LT over ECC.

These data may also be useful when evaluating a patient with ECC and presenting with prevalent or worsening FALD. Considering the higher risk of liver disease progression in patients with ECC, such patients may require exercise cardiac catheterization to screen for “occult” Fontan conduit stenosis that may not be apparent on angiography or hemodynamic indices obtained at rest. Our group demonstrated that exercise cardiac catheterization improved detection of Fontan conduit stenosis, and a favorable hemodynamic response after transcatheter stenting of such lesions.^25^^,^^26^

### Study Limitations

This is a retrospective single-center study, and it is therefore prone to selection and ascertainment bias. The clinical characteristics of the cohort may differ from that of Fontan patients at other centers, thereby limiting the generalizability of the results. We did not have liver biopsy and histopathologic data in most of our patients, and hence relied on imaging data and clinical assessment by the hepatologist.

## Conclusions

Adults who received ECC at the time of primary Fontan operation had a higher prevalence of liver cirrhosis and worse liver biomarkers at baseline, as well as a greater progression of liver dysfunction during follow-up compared to those with LT. Additionally, smaller conduit size correlated with worse liver function (higher liver biomarker scores) within the ECC group. Further studies are required to delineate the specific characteristics of the ECC that predisposes the patients to liver disease progression, and strategies to mitigate these problems.

Perspectives **COMPETENCY IN MEDICAL KNOWLEDGE:** Among patients who received TCPC at initial Fontan prevalence of severity at baseline, as well as a greater progression of liver disease during follow-up compared to patients with LT. These findings suggest the patient with ECC presenting with liver disease may require more rigorous hemodynamic assessment to screen for Fontan conduit stenosis and perhaps may require a different threshold for intervention.

**TRANSLATIONAL OUTLOOK**: Further studies are required to delineate the specific characteristics of the ECC associated with liver disease progression, and optimal strategies to mitigate these problems

## Funding support and author disclosures

The MACHD Registry is supported by the Al-Bahar research grant. Dr Egbe is supported by the 10.13039/100000050National Heart, Lung, and Blood Institute (NHLBI) grants (R01 HL158517, R01 HL160761, and R01 HL162830). All other authors have reported that they have no relationships relevant to the contents of this paper to disclose.

## References

[1] Wu F.M., Kogon B., Earing M.G. (2017). Liver health in adults with Fontan circulation: a multicenter cross-sectional study. J Thorac Cardiovasc Surg.

[2] Wu F.M., Earing M.G., Aboulhosn J.A. (2017). Predictive value of biomarkers of hepatic fibrosis in adult Fontan patients. J Heart Lung Transplant.

[3] Lindsay I., Johnson J., Everitt M.D., Hoffman J., Yetman A.T. (2015). Impact of liver disease after the Fontan operation. Am J Cardiol.

[4] Pundi K., Pundi K.N., Kamath P.S. (2016). Liver disease in patients after the Fontan operation. Am J Cardiol.

[5] Egbe A.C., Reddy Y.N.V., Khan A.R. (2018). Venous congestion and pulmonary vascular function in Fontan circulation: implications for prognosis and treatment. Int J Cardiol.

[6] Martin de Miguel I., Kamath P.S., Egbe A.C. (2023). Haemodynamic and prognostic associations of liver fibrosis scores in Fontan-associated liver disease. Heart.

[7] Miranda W.R., Kamath P.S., Jain C.C., Connolly H.C., Egbe A.C. (2023). Liver fibrosis scores are associated with resting and exercise Fontan and pulmonary artery wedge pressures: insights into FALD. Can J Cardiol.

[8] Ahmed M.H., Miranda W.R., Kamath P.S. (2024). Outcomes of esophageal varices in adults with Fontan palliation and liver cirrhosis. CJC Pediatr Congenit Heart Dis.

[9] Kim Y.Y., Lluri G., Haeffele C. (2024). Hepatocellular carcinoma in survivors after Fontan operation: a case-control study. Eur Heart J.

[10] Egbe A.C., Poterucha J.T., Warnes C.A. (2018). Hepatocellular carcinoma after Fontan operation: multicenter case series. Circulation.

[11] Elder R.W., McCabe N.M., Hebson C. (2013). Features of portal hypertension are associated with major adverse events in Fontan patients: the VAST study. Int J Cardiol.

[12] Ben Ali W., Bouhout I., Khairy P., Bouchard D., Poirier N. (2019). Extracardiac versus lateral tunnel fontan: a meta-analysis of long-term results. Ann Thorac Surg.

[13] Weixler V.H.M., Zurakowski D., Kheir J. (2020). Fontan with lateral tunnel is associated with improved survival compared with extracardiac conduit. J Thorac Cardiovasc Surg.

[14] Stewart R.D., Pasquali S.K., Jacobs J.P. (2012). Contemporary Fontan operation: association between early outcome and type of cavopulmonary connection. Ann Thorac Surg.

[15] Kisamori E., Venna A., Chaudhry H.E. (2024). Alarming rate of liver cirrhosis after the small conduit extracardiac Fontan: a comparative analysis with the lateral tunnel. J Thorac Cardiovasc Surg.

[16] Evans W.N., Acherman R.J., Mayman G.A. (2020). The rate of hepatic fibrosis progression in patients post-fontan. Pediatr Cardiol.

[17] Egbe A.C., Miranda W.R., Connolly H.M. (2025). The Mayo Adult Congenital Heart Disease (MACHD) registry and biobank. JACC Adv.

[18] Egbe A.C., Miranda W.R., Veldtman G.R., Graham R.P., Kamath P.S. (2020). Hepatic venous pressure gradient in Fontan physiology has limited diagnostic and prognostic significance. CJC Open.

[19] Egbe A.C., Miranda W.R., Anderson J.H. (2022). Determinants and prognostic implications of hepatorenal dysfunction in adults with congenital heart disease. Can J Cardiol.

[20] Miranda W.R., Kamath P.S., Jain C.C., Connolly H.C., Egbe A.C. (2023). Liver Fibrosis scores are associated with resting and exercise fontan and pulmonary artery wedge pressures: insights into FALD. Can J Cardiol.

[21] Emamaullee J., Zaidi A.N., Schiano T. (2020). Fontan-Associated liver disease: screening, management, and transplant considerations. Circulation.

[22] Emamaullee J., Khan S., Weaver C. (2021). Non-invasive biomarkers of Fontan-associated liver disease. JHEP Rep.

[23] Egbe A.C., Miranda W.R., Anderson J.H., Borlaug B.A. (2020). Hemodynamic and clinical implications of impaired pulmonary vascular reserve in the Fontan circulation. J Am Coll Cardiol.

[24] Rijnberg F.M., Westenberg J.J.M., van Assen H.C. (2022). 4D flow cardiovascular magnetic resonance derived energetics in the Fontan circulation correlate with exercise capacity and CMR-derived liver fibrosis/congestion. J Cardiovasc Magn Reson.

[25] Miranda W.R., Jain C.C., Cabalka A.K., Taggart N.W., Connolly H.M., Egbe A.C. (2022). Exercise catheterisation to unmask Fontan pathway obstruction. Can J Cardiol.

[26] Miranda W.R., Jain C.C., Burchill L.J. (2023). Correlation between Fontan pathway diameter and inferior-superior vena cava gradients in adults undergoing exercise catheterization. Circ: Cardiovasc Intervent.

